# Supplementary material for: Molecular Basis for the Involvement of Mammalian Serum Albumin in the AGE/RAGE Axis: A Comprehensive Computational Study
Source: Int J Mol Sci. 2024 Mar 11;25(6):3204. doi: 10.3390/ijms25063204 (PMC10970403; doi:10.3390/ijms25063204)
Supplement: Supplementary file 1 [file ijms-25-03204-s001.zip › File S1.pdf]

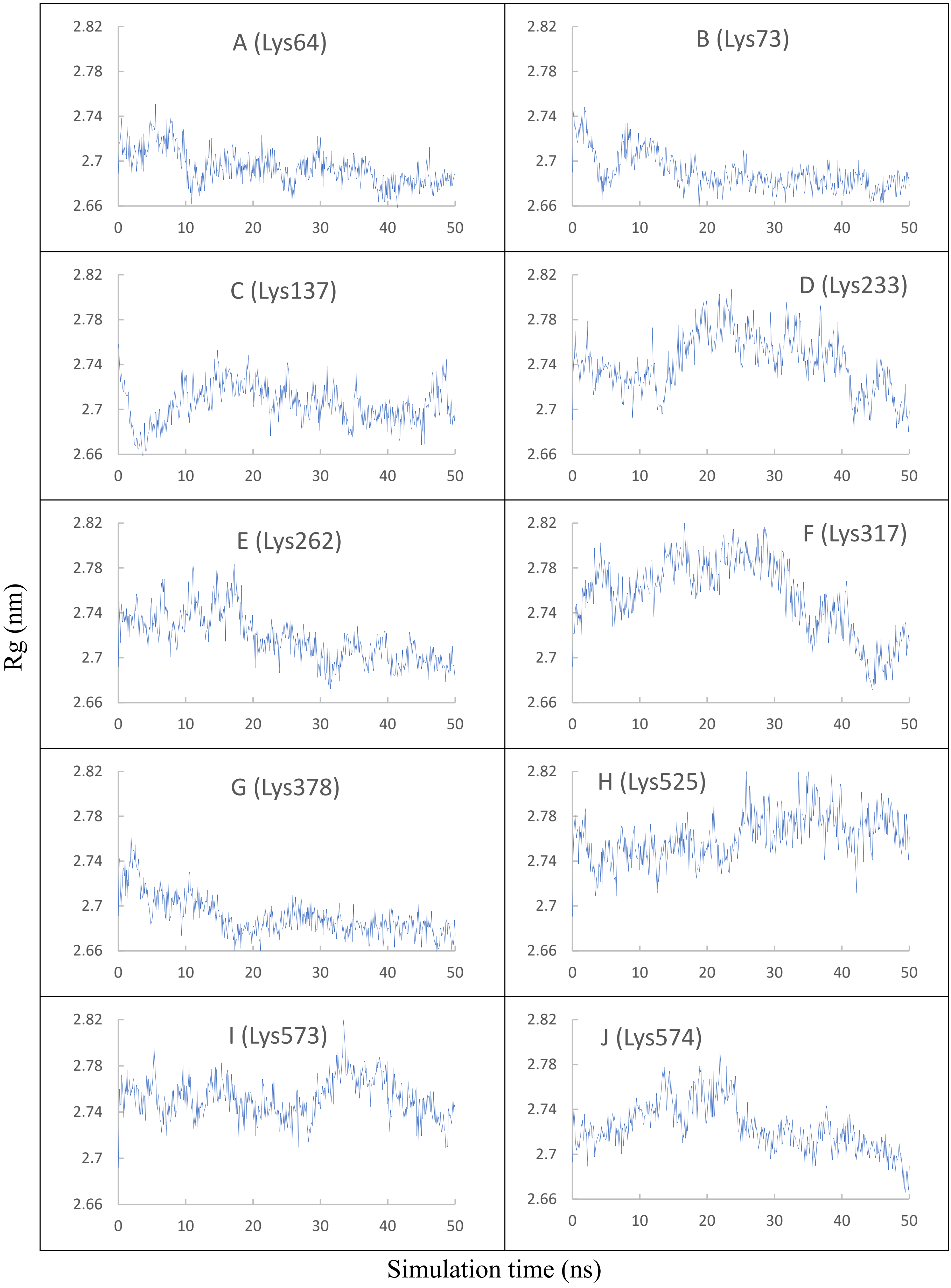

Figure S1. Radius of gyration (Rg) of human serum albumin (HSA) glycated at Lys64 (A), Lys73 (B), Lys137 (C), Lys233 (D), Lys262 (E), Lys317 (F), Lys378 (G), Lys525 (H), Lys573 (I) and Lys574 (J).

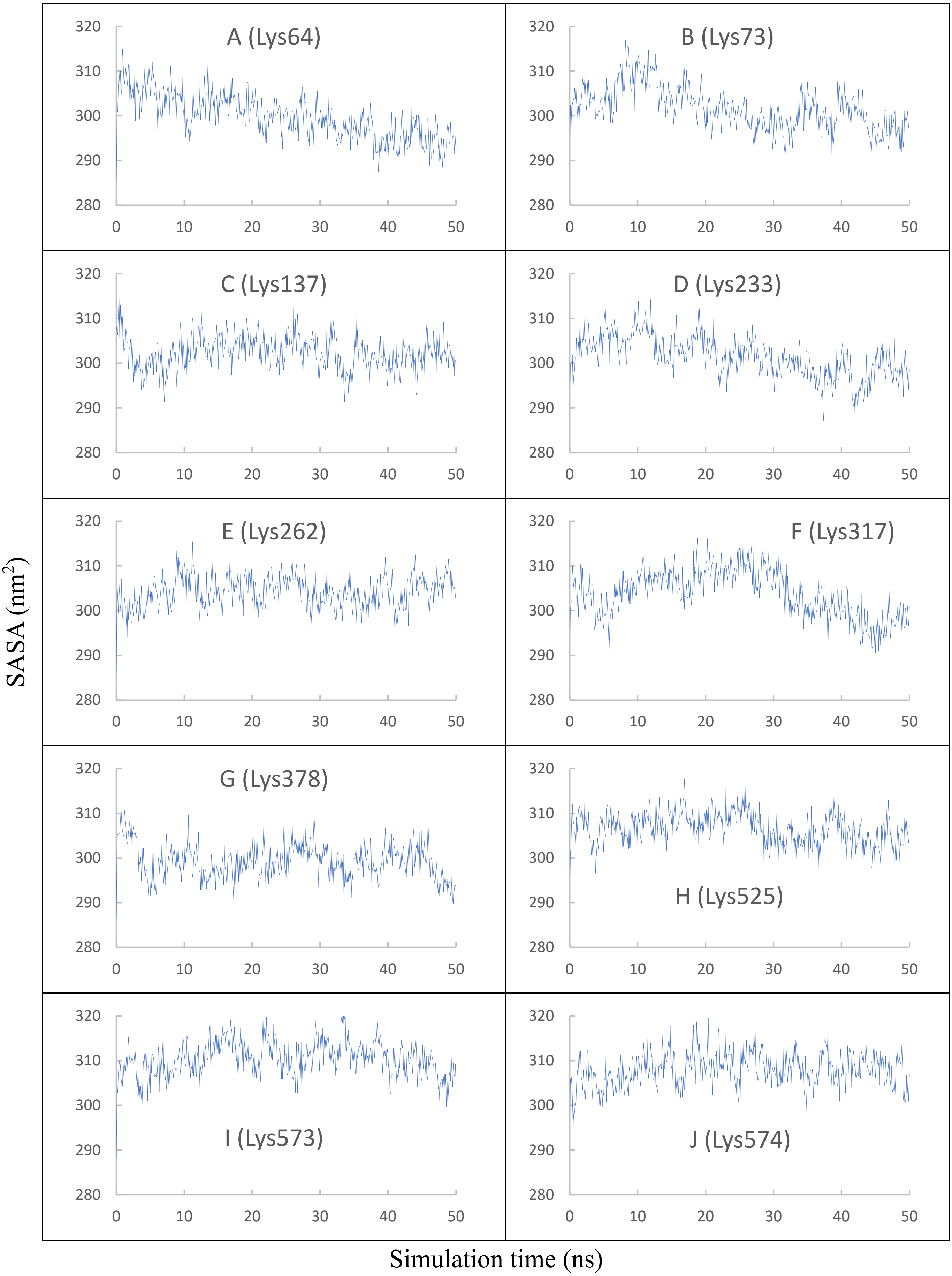

Figure S2. Solvent accessible surface area (SASA) of HSA glycated at Lys64 (A), Lys73 (B), Lys137 (C), Lys233 (D), Lys262 (E), Lys317 (F), Lys378 (G), Lys525 (H), Lys573 (I) and Lys574 (J).

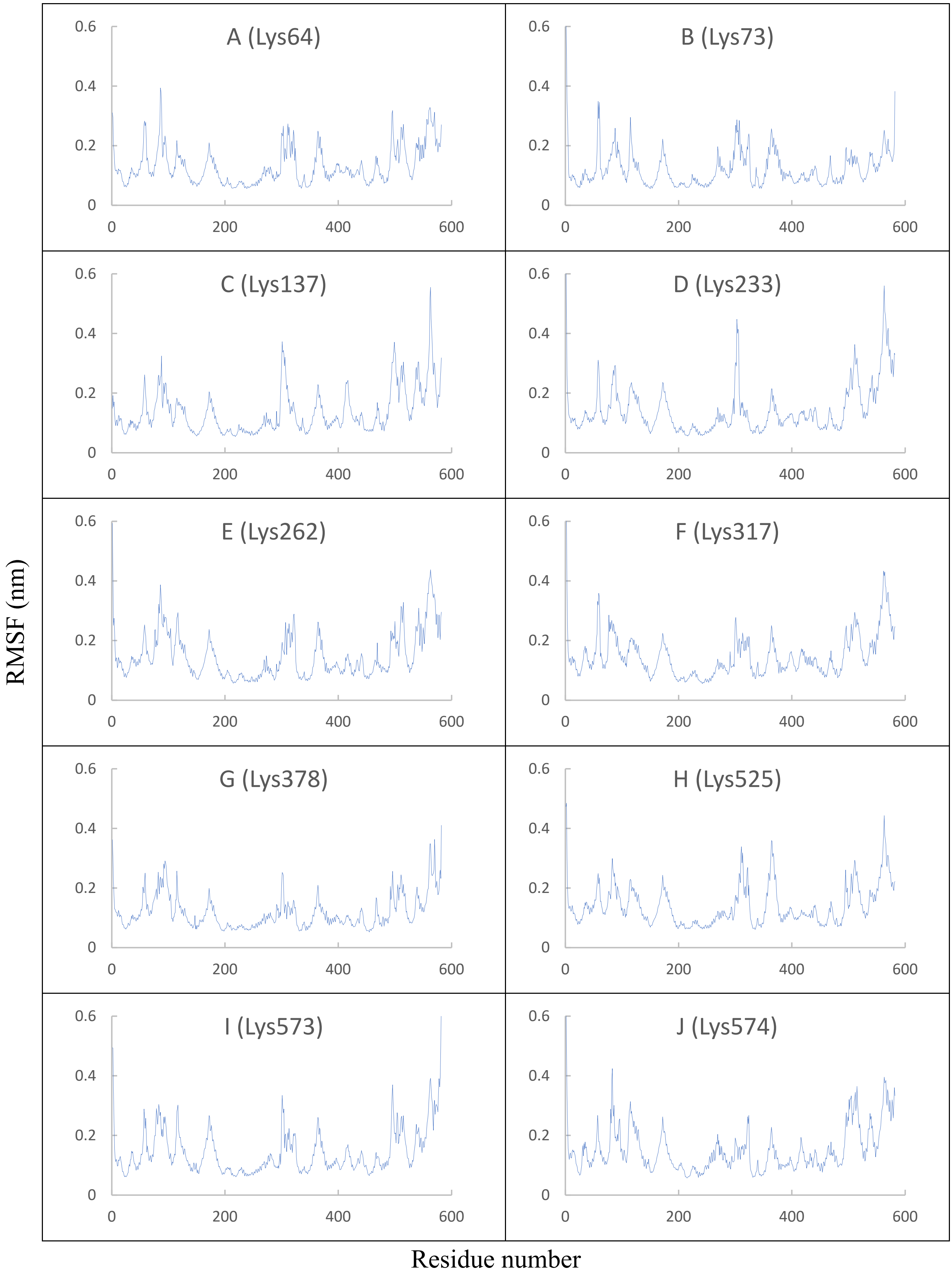

Figure S3. Root mean square fluctuation (RMSF) of Cα-atoms of HSA glycated at Lys64 (A), Lys73 (B), Lys137 (C), Lys233 (D), Lys262 (E), Lys317 (F), Lys378 (G), Lys525 (H), Lys573 (I) and Lys574 (J).

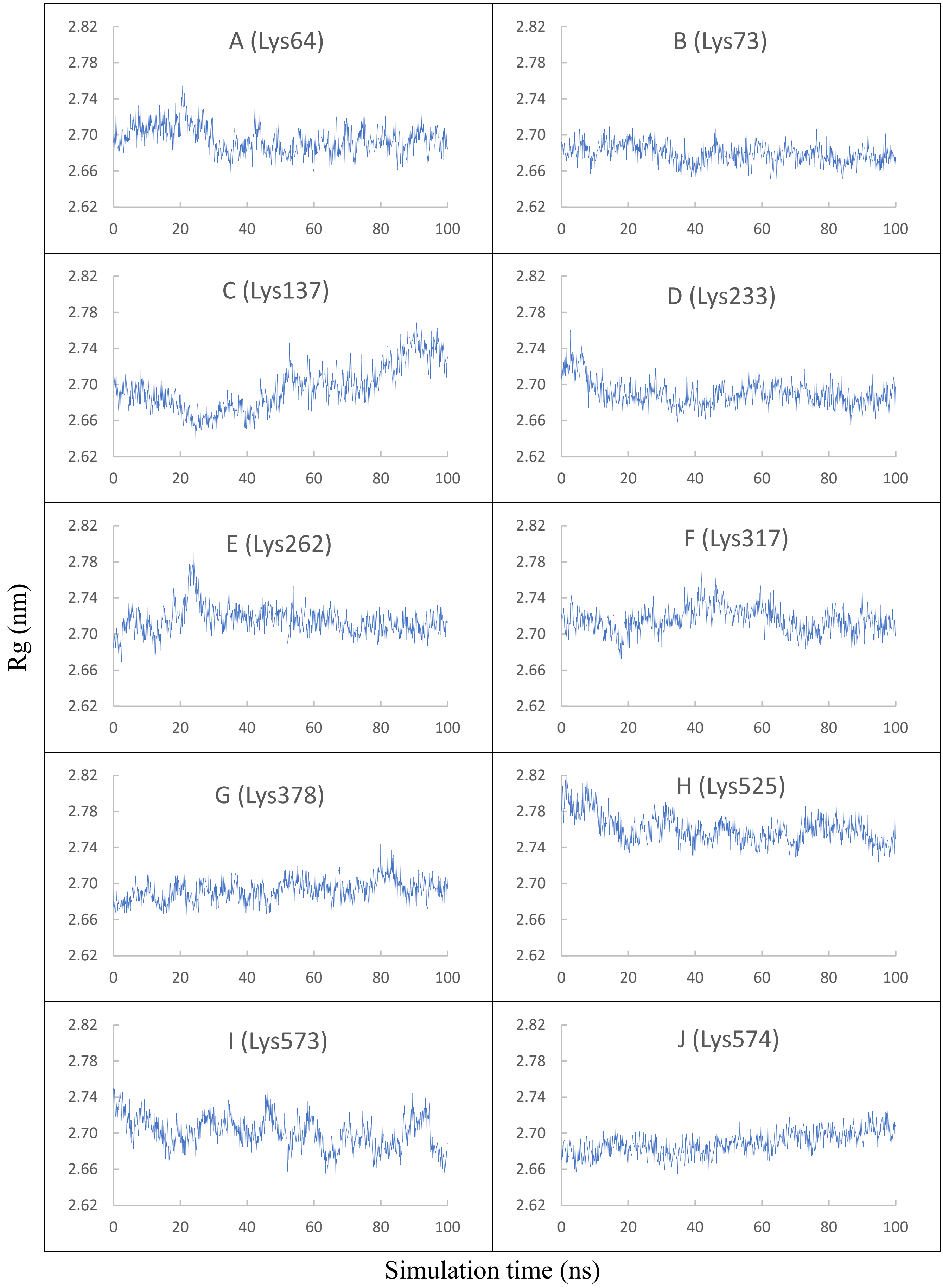

Figure S4. Rg of HSA glycated at Lys64 (A), Lys73 (B), Lys137 (C), Lys233 (D), Lys262 (E), Lys317 (F), Lys378 (G), Lys525 (H), Lys573 (I) and Lys574 (J) in complex with V-domain of receptor for advanced glycation end products (RAGE).

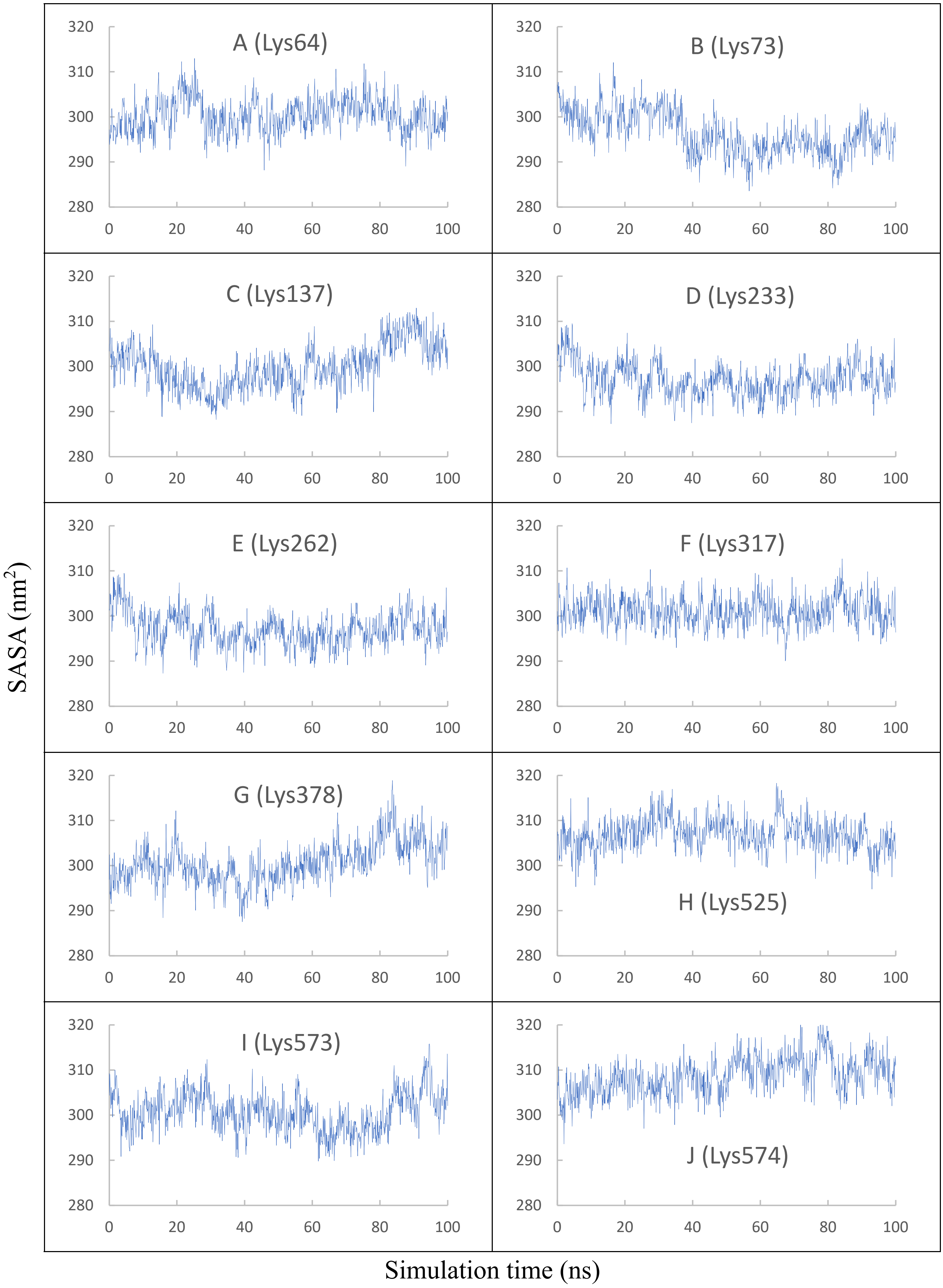

Figure S5. SASA of HSA glycated at Lys64 (A), Lys73 (B), Lys137 (C), Lys233 (D), Lys262 (E), Lys317 (F), Lys378 (G), Lys525 (H), Lys573 (I) and Lys574 (J) in complex with V-domain of RAGE.

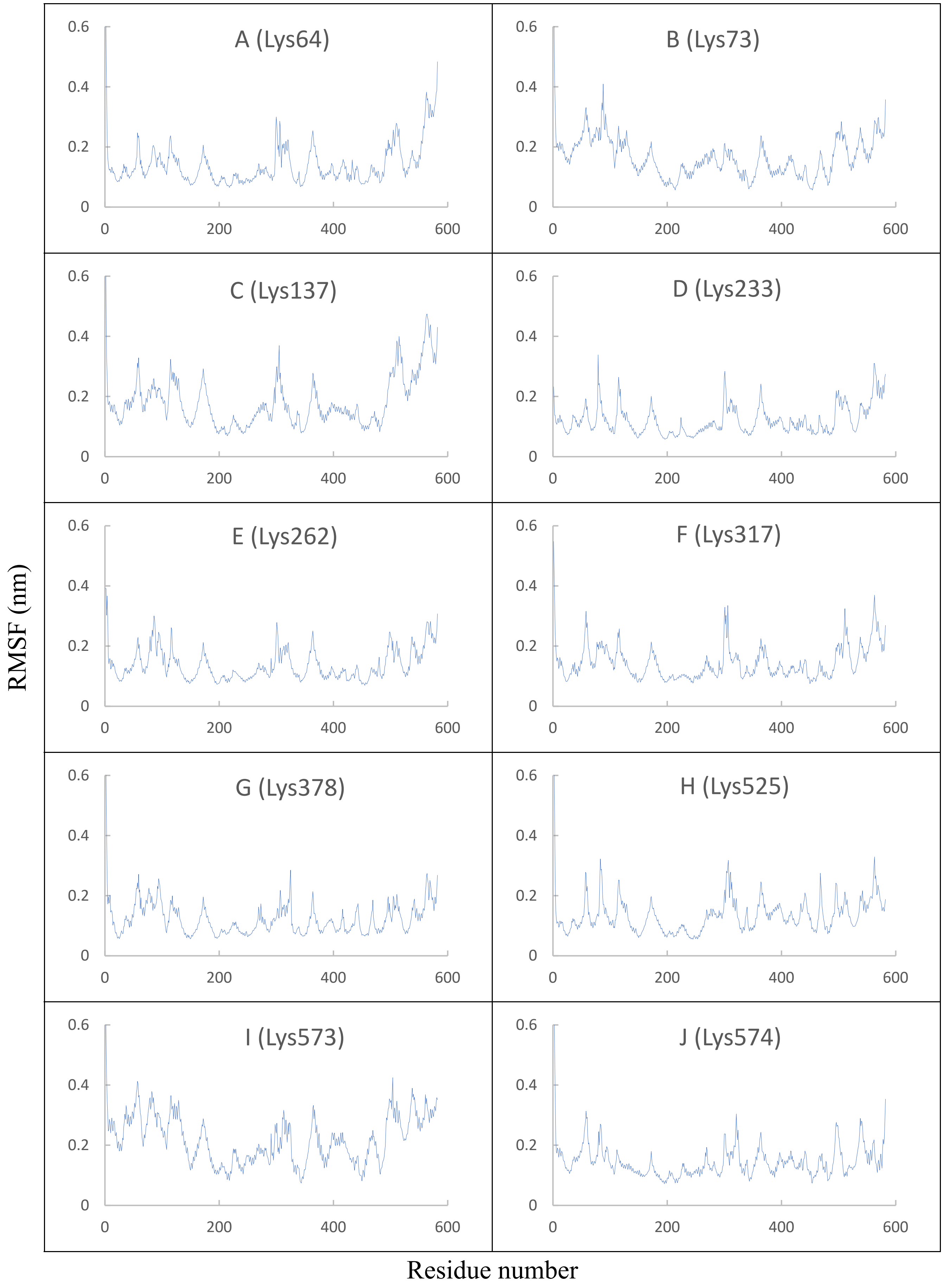

Figure S6. RMSF of Cα-atoms of HSA glycated at Lys64 (A), Lys73 (B), Lys137 (C), Lys233 (D), Lys262 (E), Lys317 (F), Lys378 (G), Lys525 (H), Lys573 (I) and Lys574 (J) in complex with V-domain of RAGE.

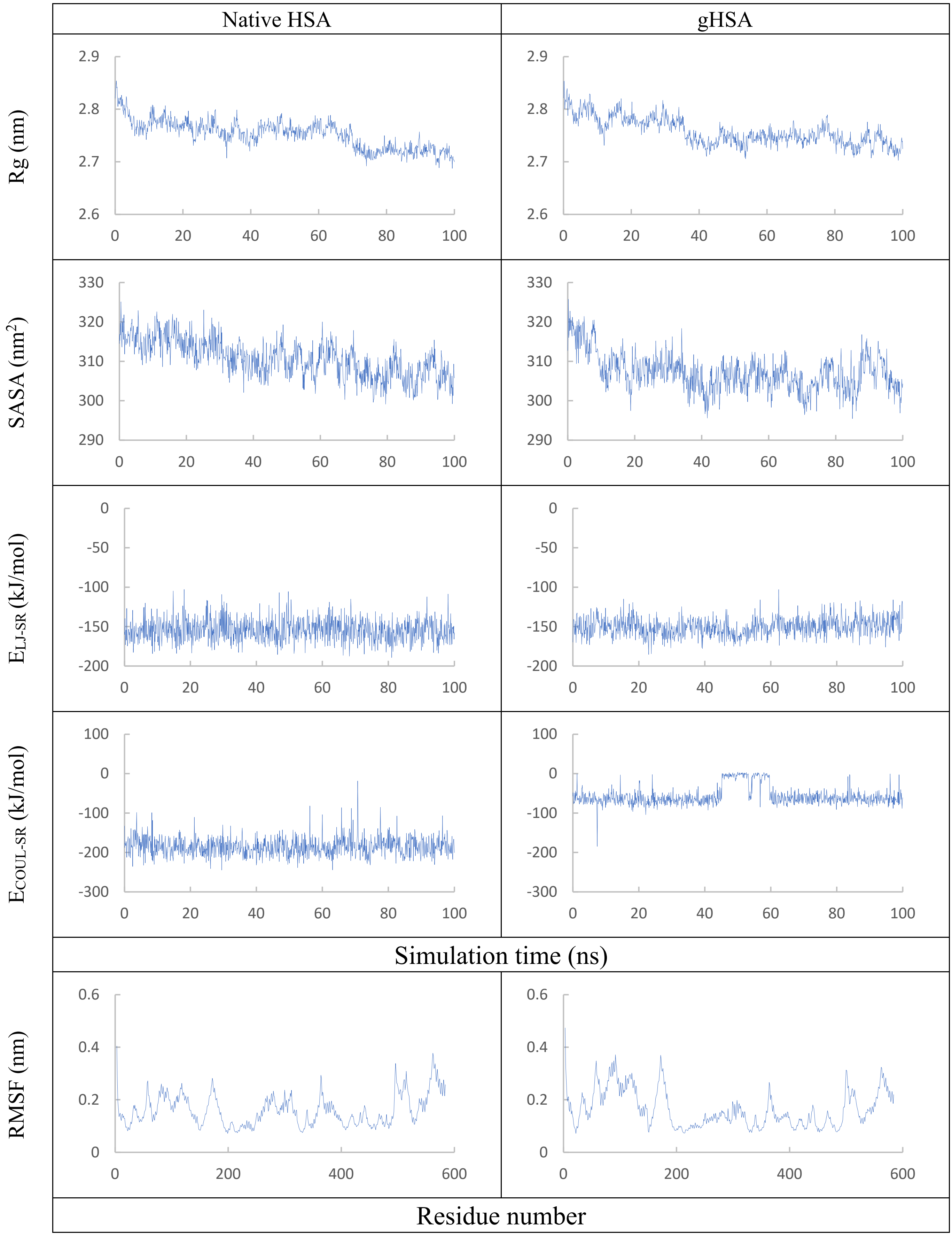

Figure S7. Conformation and energy characteristics of the complexes of oleic acid (OLA) with fatty acid binding site FA5 of native albumin (HSA) and albumin glycated at Lys525 to carboxymethyl-lysine (gHSA). Rg, radius of gyration of HSA/gHSA; SASA, solvent accessible surface area of HSA/gHSA; $E_{LJ\text{-}SR}$, short-range Lennard-Jones potential of interaction between OLA and HSA/gHSA; $E_{COUL\text{-}SR}$, short-range Coulomb potential of interaction between OLA and (g)HSA; RMSF, root mean square fluctuation of Cα-atoms of HSA/gHSA.
